# Psychological and Physical Health Outcomes in Adults With Craniosynostosis

**DOI:** 10.1177/10556656211059966

**Published:** 2021-12-06

**Authors:** Nicola Marie Stock, Bruna Costa, Karen Wilkinson-Bell, Laura Culshaw, Anna Kearney, Wendy Edwards

**Affiliations:** 1 Centre for Appearance Research, University of the West of England, Coldharbour Lane, Bristol, United Kingdom; 2 Headlines Craniofacial Support, St Albans, Hertfordshire, United Kingdom; 3 Alder Hey Craniofacial Unit, Alder Hey Children's NHS Foundation Trust, Liverpool, United Kingdom

**Keywords:** craniosynostosis, adult, mental health, physical health, treatment

## Abstract

**Objectives:**

Within current research, little is known about the long-term outcomes of craniosynostosis. A priority-setting exercise by UK charity Headlines Craniofacial Support identified 2 key questions in this area: (1) What are the long-term physical and psychological effects for individuals with syndromic and non-syndromic craniosynostosis? and (2) Are individuals with craniosynostosis likely to suffer from mental health difficulties, or are they more resilient? The aim of the current study was to conduct an initial investigation of these priority questions.

**Methods:**

A comprehensive UK-wide survey consisting of 9 standardized psychological outcome measures and open-ended questions was distributed online. Thirty-six eligible adults (69.4% female) with a mean age of 30.8 years responded to the survey. Participants reported having single suture craniosynostosis (27.8%) or syndromic craniosynostosis (52.8%), with 19.4% being unsure of their diagnosis. Sample means were compared to published norms using independent samples *t* tests. Qualitative responses were analysed using inductive content analysis.

**Results:**

Compared to the general population, participants reported significantly less favorable scores related to appearance concerns, attachment in adult relationships, anxiety, optimism, and resilience. Self-worth, depression, and social anxiety scores were similar to norms. Qualitative responses provided additional insight into participants’ satisfaction with appearance, physical health, medical treatment, employment, relationships, and recurrence risks. Few participants had accessed psychological support.

**Discussion:**

This preliminary study illustrates the potential long-term implications for individuals with craniosynostosis. Improved treatment protocols are needed to address physical health concerns in adulthood, while dedicated psychological resources are necessary to promote emotional well-being, social confidence, and resilience.

## Introduction

Craniosynostosis is a relatively rare and complex condition, in which the clinical presentation can vary considerably (Kajdic et al., 2018). It occurs when 2 or more of the cranial plates fuse prematurely in utero and affects approximately 1 in every 2000 live births (McCarthy et al., 2012). Syndromic forms of craniosynostosis (including Apert, Pfeiffer, and Crouzon syndromes) are much rarer. Depending on the severity of the condition, early surgical intervention and ongoing multidisciplinary treatment throughout childhood may be necessary. Craniosynostosis can result in the individual having a visible facial difference, alongside a range of medical challenges (Kajdic et al., 2018).

In recent years, the potential psychological impacts of congenital craniofacial conditions have become more widely recognized within both clinical practice and research (Feragen and Stock, 2017). Nonetheless, the literature to date has focused predominantly on the early years, examining the psychological impacts of craniosynostosis on children born with the condition and their parents. However, studies published in the last few years have also begun to explore the longer-term outcomes of those affected, using both quantitative and qualitative methods to examine quality of life in adulthood (Feragen and Stock, 2017).

While some studies have identified a range of difficulties faced by adults with syndromic and non-syndromic craniosynostosis, including depressed mood, poor physical health, and fewer friends and romantic partners compared to controls (Stavropoulos et al., 2011; Tovetjärn et al., 2012; Fischer et al., 2014), others have found adults with craniosynostosis to report equal or superior quality of life to the general population (Lloyd et al., 2016; Mazzaferro et al., 2018; Salokorpi et al., 2019). Correspondingly, a recent review of the craniofacial literature (Feragen and Stock, 2017) reported that findings in regard to psychological adjustment to craniofacial conditions were largely inconclusive. The authors of the review concluded that, rather than assessing broad quality of life, researchers should seek to examine individual domains of psychological well-being. The authors also recommended an increase in the use of qualitative methods to elicit a more holistic understanding of what it is like to live with a craniofacial condition, and a need to investigate the positive, as well as the negative impacts. Finally, collection of samples including both syndromic and non-syndromic cases was recommended, so that any differences and/or commonalities across diagnostic subtypes can be explored.

In the United Kingdom (UK), care for those affected by craniosynostosis is delivered by 5 supra-regional specialist units, with additional community-based support offered by Headlines Craniofacial Support (Headlines), a UK-wide organization that became a registered charity in 1996. Over the years, Headlines has developed a range of services, including a helpline, a welfare fund, information leaflets, family events, fundraising activities, e-newsletters, and a biennial conference. However, Headlines currently offers little for adults. Similarly, the 5 specialist units did not exist when the majority of today's adults were growing up, and are not currently commissioned to provide care throughout adulthood. As such, the current adult population has received comparatively less support.

In 2017, Headlines carried out a wide-ranging consultation with their members and with health professionals working in the field to produce a list of priority research questions. Two of these priorities focused on long-term outcomes: (1) What are the long-term physical and psychological effects for individuals with syndromic and non-syndromic craniosynostosis? and (2) Are individuals with craniosynostosis likely to suffer from mental health difficulties, or are they more resilient? The aim of the current study was to conduct an initial exploration of these 2 priority research questions via the administration of a comprehensive UK-wide survey among adults with syndromic and non-syndromic craniosynostosis.

## Methods

### Design

Ethical approval to conduct the study was obtained from the Faculty Research Committee at the University of the West of England. An online survey (Table 1) was designed using the platform Qualtrics, drawing upon current craniofacial literature (eg, Feragen and Stock, 2017) and clinical and research expertise. The survey consisted of 9 standardized patient-reported outcome measures, chosen for their psychometric properties, clinical utility, the availability of general population norms, and for consistency with related studies (see Stock et al., 2020a for a review). Topics of interest included satisfaction with appearance, social experiences, symptoms of anxiety and depression, physical health, self-worth, and resilience. Demographic data were also collected (Table 2), alongside open-ended questions for participants to provide more detail if they wished. Clinical psychologists from the 4 specialist units in England and 10 adults born with craniosynostosis provided feedback on the study design and draft materials. A version of the survey was also administered to parents of children born with craniosynostosis, the findings of which are detailed in separate papers (*under review*).

**Table 1.** Survey Outline.

Survey section	Questions included	Measure reference	Comparison group reference
Demographic Information	Age	–	Office for National Statistics (2018)
	Gender		
	Ethnic group		
	Highest educational qualification		
	Current employment status		
	Marital status		
	Place of birth		
	Condition type		
	Family history of craniosynostosis		
Emotional Well-being	Connor-Davidson Resilience Scale (CD-RISC10)	Connor & Davidson, 2003	Connor & Davidson, 2003
	Revised Life Orientation Test (LOT-R)	Scheier et al., 1994	Bredal et al., 2017
	Hospital Anxiety and Depression Scale (HADS)	Zigmond & Snaith, 1983	Crawford et al., 2001
	Self-Perception Profile for Adults (SPP-AA; Global Self-Worth subscale)	Messer & Harter, 2012	Messer & Harter, 2012
	[Open-ended] Is there anything else you would like to share about your emotional well-being?	–	–
Relationships	Self-Perception Profile for Adults (SPP-AA; Social Competence subscale)	Messer & Harter, 2012	Messer & Harter, 2012
	Mini Social Phobia Inventory (Mini-SPIN)	Connor et al., 2000	Seeley-Wait et al., 2009
	Self-Perception Profile for Adults (SPP-AA; Intimate Relationships subscale)	Messer & Harter, 2012	Messer & Harter, 2012
	Revised Adult Attachment Scale (R-AAS)	Collins, 1996	Collins, 1996
	[Open-ended] Is there anything else you would like to share about your relationships?	–	–
Having Children	[Yes/No] Do you currently have children?	–	–
	[Yes/No] Did/does the possibility of having a child born with craniosynostosis cause you concern?	–	–
	[Open-ended] Is there anything else you would like to share about having children?	–	–
Education and Employment	[Open-ended] What has been your experience of job seeking?	–	–
	[Open-ended] Is there anything else you would like to share about your educational and/or employment experiences?	–	–
Appearance Satisfaction	Body Esteem Scale for Adolescents and Adults (BES-AA; Appearance Evaluation subscale)	Mendelson et al., 2001	Mendelson et al., 2001
	Fear of Negative Appearance Evaluation Scale (FNAES)	Lundgren et al., 2004	Lundgren et al., 2004
	Perceived Sociocultural Pressures Scale (PSPS)	Diedrichs et al., 2015	Diedrichs et al., 2015
	[Open-ended] Is there anything else you would like to share about your appearance?	–	–
Physical Health	[No/Yes Occasionally/Yes Frequently] Do you have any of ongoing physical health difficulties with the following (eg, speech, hearing, fatigue)?	–	–
	[Open-ended] If yes, please describe the impact these difficulties have on you?	–	–
	[Open-ended] Is there anything else you would like to share about your physical health?	–	–
Healthcare Experiences	[Open-ended] What has been your experience of healthcare as an adult?	–	–
	[Yes/No] Have you received psychological support for challenges related to craniosynostosis?	–	–
	[Open-ended] Is there anything else you would like to share about your healthcare experiences?	–	–

**Table 2.** Participant Characteristics.

		% of sample	UK Census Data (%)
Age			
	Mean: 30.83		
	SD: 12.38		
Gender			
	Female	69.4	50.9
	Male	27.8	49.1
	Prefer not to say	2.8	–
Ethnicity			
	White British	91.7	86.0
	Other	8.4	14.0
Employment status			
	Employed	75	58.7
	Unemployed	8.3	4.6
	Retired/Student/Disabled	16.7	36.7
Education level			
	No qualifications	2.8	22.7
	Below degree level	44.4	50.1
	Degree level or above	44.5	27.2
	Prefer not to say	8.3	–
Marital Status			
	Single/never married	62.9	34.6
	Married/partnered	25.0	51.1
	Divorced/separated	11.1	8.0
	Widowed	–	6.4
Parenthood			
	No children	75.0	18.0
	One or more children	25.0	82.0
Diagnosis			
	Non-syndromic single suture craniosynostosis	27.8	–
	Crouzon syndrome	27.8	–
	Apert syndrome	16.7	–
	Saethre-Chotzen syndrome	5.6	–
	Don’t know/Not sure	19.4	–
Known Family History of Craniosynostosis			
	Yes	19.4	–
	No	80.6	–

### Procedure

A link to the online survey was distributed on relevant websites, social media, and e-newsletters inviting adults with craniosynostosis to take part. Before proceeding to the survey itself, participants were asked to read the Participant Information and Privacy Notice, and to indicate their consent to participate. Participants were eligible if they were born with craniosynostosis, aged 16 years or older, living in the UK, and understood written English well enough to provide informed consent. Both individuals with syndromic and non-syndromic craniosynostosis were invited to participate, in order to be inclusive (Feragen et al., 2014) and in an attempt to address the research priority questions in full. The survey was launched in October 2019 and remained open to responses until April 2020.

### Analysis

A review, verification and validation of quantitative data was undertaken prior to analysis. To examine possible differences across the sample according to syndromic status, independent samples *t* tests were carried out. In 7 cases, the syndromic status could not be determined and these participants were excluded from the exploratory analysis.

Sample means were generated and compared to published general population data obtained from Europe and the United States (Table 1) using independent samples *t* tests. Cohen's *d* was also calculated, whereby a value of 0.2–0.5 represents a small effect, 0.5–0.8 represents a medium effect, and 0.8 or more represents a large effect (Cohen, 1988). Pearson's Correlation was used to assess the relationship between outcome measures. For statistically significant correlations, *r* values of approximately 0.1 in magnitude are considered to represent a small effect, 0.3 to represent a medium effect, and 0.5 to represent a large effect (Cohen, 1988).

Qualitative data were analysed independently using inductive content analysis (Hsieh and Shannon, 2005) by the first and second authors, who are trained in qualitative methods. First, the data were read and re-read, to establish an overall picture of the data. Common codes were then inductively grouped together in an iterative process and discussed until full agreement was reached. Finally, frequency counts were calculated. Preliminary findings were shared with patient representatives and clinical psychologists, and their feedback was integrated into the final paper.

## Results

Thirty-six eligible adults born with craniosynostosis responded to the survey, with between 31 and 34 participants completing each of the standardized measures. Participants had a mean age of 30.4 years (SD 12.4) and were predominantly White British (91.7%) and female (69.4%). Compared to National Census data (Office for National Statistics, 2018; Table 2), the current sample had slightly raised levels of unemployment, despite being well educated on average. Participants were also more likely than the general population to be single and/or without children. The most common diagnoses reported were single suture craniosynostosis (27.8%) and Crouzon syndrome (27.8%). Most participants did not have a known family history of craniosynostosis.

No differences were found between adults with syndromic and non-syndromic diagnoses, with one exception, where adults with non-syndromic craniosynostosis (*M* = 3.19, *SD* = 0.273) reported significantly lower self-perceived competence in intimate relationships than adults with syndromic craniosynostosis (*M* = 3.50, *SD* = 0.364), *t*(25) = −2.218, *P* = .036, *d* = 0.964.

In comparison to the general population (Table 3), participants reported significantly more fear of negative appearance evaluation, poorer body image, and more pressure to conform to societal appearance ideals. Adults with craniosynostosis also reported significantly less comfort in adult relationships compared with the general population. Levels of optimism and resilience were significantly lower in adults with craniosynostosis compared to general population norms, and symptoms of anxiety were significantly elevated. However, levels of social anxiety, self-perceived social competence, depression, and self-worth were similar to the general population. Despite describing a range of ongoing challenges, only one participant (3.2%) reported having accessed psychological support as an adult.

**Table 3.** Comparisons to Normative Data.

Outcome measure	Sample mean (SD)	Comparative mean (SD)	Independent samples *t* test	Cohen's *d*
Body Esteem Scale for Adolescents and Adults	*n* = 31 1.65 (1.05)	*n* = 133 2.5 (0.7)	*t*(36) = −4.30, *p* < .001	0.954
Fear of Negative Appearance Evaluation Scale	*n* = 31 3.38 (1.06)	*n* = 201 2.13 (1.20)	*t*(42) = 6.601, *p* < .001	1.104
Perceived Sociocultural Pressures Scale	*n* = 30 1.66 (0.53)	*n* = 1531 1.44 (0.63)	*t*(30) = 2.23, *p* < .05	0.378
Self-Perception Profile for Adults (Social Competence subscale)	*n* = 32 3.30 (0.64)	*n* = 215 3.13 (0.64)	*t*(40) = 1.40, *p* = .168	–
Mini Social Phobia Inventory	*n* = 32 2.17 (1.05)	*n* = 56 1.8 (1.6)	*t(*84) = 1.30, *p* = .196	–
Self-Perception Profile for Adults (Intimacy subscale)	*n* = 32 3.37 (0.38)	*n* = 215 3.15 (0.66)	*t*(63) = 2.72, *p* < .01	0.408
Revised Adult Attachment Scale (Close subscale)	*n* = 32 3.21 (0.85)	*n* = 68 4.02 (0.87)	*t*(62) = 4.41, *p* < .001	0.914
Revised Adult Attachment Scale (Depend subscale)	*n* = 32 2.89 (0.87)	*n* = 68 3.69 (0.88)	*t*(61) = −4.28, *p* < .001	0.915
Revised Adult Attachment Scale (Anxiety subscale)	*n* = 32 3.50 (1.16)	*n* = 68 2.53 (1.05)	*t*(55) = 4.01, *p* < .001	0.875
Hospital Anxiety and Depression Scale (Anxiety subscale)	*n* = 32 9.25 (4.42)	*n* = 1792 6.14 (3.76)	*t*(31) = 3.95, *p* < .001	0.758
Hospital Anxiety and Depression Scale (Depression subscale)	*n* = 32 4.22 (3.91)	*n* = 1792 3.68 (3.07)	*t*(31) = 0.78, *p* = .443	–
Self-Perception Profile for Adults (Global Self-Worth subscale)	*n* = 32 3.32 (0.81)	*n* = 215 3.18 (0.55)	*t*(35) = 0.95, *p* = .351	–
Revised Life Orientation Test	*n* = 34 11.47 (4.82)	*n* = 1792 17.2 (3.0)	*t*(33) = −6.91, *p* < .001	1.428
Connor-Davidson Resilience Scale	*n* = 34 26.47 (7.35)	*n* = 458 32.1 (5.8)	*t*(36) = −4.37, *p* < .001	0.850

Pearson's Correlation analyses found that most outcomes were correlated to varying degrees (*r* = 0.042 to 0.838; Table 4). The most common physical complaints among adults with craniosynostosis were fatigue (51.6%), hearing difficulties (45.2%), vision problems (41.9%), migraines (38.7%), and dental issues (35.5%).

**Table 4.** Associations Between Outcome Measures.

	BES-AA	CD-RISC10	FNAES	HADS-A	HADS-D	LOT-R	Mini-SPIN	R-AAS Close	R-AAS Depend	R-AAS Anxiety	SPP-Ad Global Self-Worth	SPP-Ad Social Competence	SPP-Ad Intimacy	PSPS
BES-AA		*r*(31) = 0.481**	*r*(31) = −0.688***	*r*(31) = −0.629***	*r*(31) = −0.659***	*r*(31) = 0.777***	*r*(31) = −0.664***	*r*(31) = 0.342, *P* = .060	*r*(31) = 0.432*	*r*(31) = −0.399*	*r*(31) = 0.838***	*r*(31) = 0.548***	*r*(31) = 0.377*	*r*(30) = −0.591***
CD-RISC10			*r*(31) = −0.366*	*r*(32) = −0.504**	*r*(32) = −0.768***	*r*(34) = 0.494**	*r*(32) = −0.496**	*r*(32) = 0.360*	*r*(32) = 0.507**	*r*(32) = −0.478**	*r*(32) = 0.652***	*r*(32) = 0.609***	*r*(32) = 0.160 *P* = .381	*r*(30) = −0.546**
FNAES				*r*(31) = 0.610***	*r*(31) = 0.609***	*r*(31) = −0.663***	*r*(31) = 0.556***	*r*(31) = −0.390*	*r*(31) = −0.532**	*r*(31) = 0.554***	*r*(31) = −0.734***	*r*(31) = −0.444*	*r*(31) = −0.311, *P* = .088	*r*(30) = 0.580***
HADS-A					*r*(32) = 0.583***	*r*(32) = −0.610***	*r*(32) = 0.466**	*r*(33) = −0.350, *P* = .050	*r*(32) = −0.466**	*r*(32) = 0.538**	*r*(32) = −0.703***	*r*(32) = −0.606***	*r*(32) = −.0.042, *P* = .820	*r*(30) = 0.560***
HADS-D						*r*(32) = −0.628***	*r*(32) = 0.589***	*r*(32) = −0.505**	*r*(32) = −0.623***	*r*(32) = 0.373*	*r*(32) = −0.727***	*r*(32) = −0.672***	*r*(32) = −0.305, *P* = .090	*r*(30) = 0.729***
LOT-R							*r*(32) = −0.612***	*r*(32) = 0.330, *P* = .066	*r*(32) = 0.530**	*r*(32) = −0.566***	*r*(32) = 0.833***	*r*(32) = 0.446*	*r*(32) = 0.238, *P* = .190	*r*(30) = −0.580***
Mini-SPIN							* *	*r*(32) = −0.352*	*r*(32) = −0.396*	*r*(35) = 0.496**	*r*(32) = . −0.651***	*r*(32) = −0.629***	*r*(32) = −0.392*	*r*(30) = 0.598***
R-AAS Close							* *	* *	*r*(32) = 0.639***	*r*(32) = −0.503**	*r*(32) = 0.308, *P* = .086	*r*(32) = 0.702***	*r*(32) = 0.652***	*r*(30) = −0.200, *P* = .288
R-AAS Depend							* *	* *	* *	*r*(32) = −0.706***	*r*(32) = 0.504**	*r*(32) = 0.509**	*r*(32) = 0.294, *P* = .102	*r*(30) = −0.509**
R-AAS Anxiety							* *	* *	* *	* *	*r*(32) = −0.529**	*r*(32) = −0.555***	*r*(32) = −0.065, *P* = .722	*r*(30) = 0.452*
SPP-Ad Global Self-Worth							* *	* *	* *	* *	* *	*r*(32) = 0.563***	*r*(32) = 0.283, *P* = .117	*r*(30) = −0.690***
SPP-Ad Social Competence							* *	* *	* *	* *	* *	* *	*r*(32) = 0.384*	*r*(30) = −0.637***
SPP-Ad Intimacy							* *	* *	* *	* *	* *	* *	* *	*r*(30) = 0.025, *P* = .895

*Significant at *P* < .05; **Significant at *P* < .01; ***Significant at *P* < .001.

Common themes, codes, exemplar quotes, and frequency counts extracted from the qualitative data are provided in Table 5. Participants described how their appearance impacted on their self-esteem, romantic relationships, employment opportunities, and identity. They also outlined how having a difference that was visible to other people could evoke unwanted reactions, and a desire to hide/change their appearance. Social impacts of craniosynostosis included difficulties in feeling accepted by others, initiating new relationships, overcoming social hurdles, and explaining the condition to others. Participants also reported experiencing symptoms of social anxiety. In terms of treatment, participants reported challenges with the transition to adult care, a lack of follow-up from their craniofacial team during adulthood, and a desire for more information and psychological support. A lack of awareness of craniosynostosis among non-specialist health professionals was identified, alongside difficulties obtaining treatment referrals, important connections between symptoms being missed, and insensitive language being used during consultations. Some participants had not been made aware of their condition until adulthood. Participants also reported a cumulative effect of repeated medical stress on their mental well-being. Another theme identified in the qualitative data was employment, with different views on how much craniosynostosis had impacted participants in terms of finding work. Participants identified the emotional impact of job seeking, and felt their condition affected them regularly while in work. For the majority of participants, recurrence risk was a concern when considering starting a family, yet others did not feel this was a significant deterrent. Finally, participants offered advice to others with craniosynostosis. These were all positively framed in nature and reflective of adaptive self-care practices.

**Table 5.** Content Analysis of Qualitative Data.

Themes	Codes	Number of references	Exemplar quotes
Appearance	Impact on self-esteem	29	“I am very self-conscious of my appearance”
	Other people's reactions	22	“Every single day someone has something to say about my face”
	Impact on romantic relationships	11	“Girls don’t find me attractive”
	Desire to change appearance	8	“I wish I looked normal”
	Impact on employment opportunities	5	“People would prefer to hire someone without a facial disfigurement”
	Hiding affected features	3	“Because of the scars on my head…I am absolutely terrified of going bald and I often wear a hat”
	Impact on identity	3	“Some people say I don’t look like I have Crouzon's so I must have a mild case. This can feel very dismissive and hurtful as I’ve had over 30 surgeries to correct my appearance”
Social Experiences	Being accepted by others	30	“It's been hard to fit in… I’ve felt like an outsider”
	Social anxiety	19	“Socially I feel very anxious, especially around people I don’t know well”
	Difficulty initiating relationships	16	“I find it very daunting…dating and friendships are difficult”
	Overcoming social hurdles	15	“I have to make a conscious effort to be open and friendly”
	Difficulty explaining condition to others	7	“It's hard to…explain to people what it is and how I really feel about it”
Treatment Experiences in Adulthood	Increased awareness needed among non-specialists	25	“It would make me feel more secure and well looked after if my GP knew more about my condition than I do!”
	No follow-up during adulthood	18	“There has been no follow-up…I wouldn’t know who to speak to about any concerns”
	Psychological support needed in adulthood	15	“Psychological support to cope with physical health problems, upcoming treatment and other aspects of my condition that I find difficult”
	Difficulties accessing appropriate care	10	“I’ve had problems getting referrals”
	Long-term effects of medical stress	8	“I have been traumatised by my medical experiences”
	No connections between symptoms made	5	“Health professionals don’t consider the causes or connections between symptoms”
	Craniosynostosis undiagnosed until later life	5	“I wasn’t aware I had the condition… It only came to light when I had my first child”
	More information needed	4	“There doesn’t seem to be much information available for adults”
	Transition to adult care requires improvement	3	“Better support to make my own healthcare decisions”
	Insensitive language used by health professionals	3	“A lot of doctors refer to my ‘deformities’ which is upsetting to hear”
Employment	Difficulties gaining employment	17	“I have qualifications and experience but feel I am not being employed due to my disability”
	No difficulties gaining employment	12	“I’ve been in regular and satisfying employment and built a fulfilling and successful career”
	Emotional impact of job seeking	8	“I have found it very stressful and have wanted to give up”
	Physical health impacts	8	“I regularly have bad migraines…it affects my potential to perform well at work”
	Treated differently when in work	2	“I always get shipped off to Occupational Health…I feel judged and pitied”
Starting a Family	Recurrence risk is a cause of concern	29	“I wouldn’t want to put my children or grandchildren through what I’ve been through”
	Recurrence risk is not a deterrent	7	“I don’t think it would put me off… With the right support I think I could cope”
Advice to Others	Seek social support	9	“Don’t care too much about what other people think… Surround yourself with people who make you feel good”
	Be confident and proud of who you are	8	“Keep your head high”
	Find a purpose	7	“Set yourself some ambitions and achieve something regularly… Live your life to the fullest you can”
	Advocate for yourself	7	“Seek information and advice… Ask for help”
	Find the positives	6	“Embrace your condition… There are strengths and gifts in this seemingly cruel thing”
	Believe everything will be OK	4	“You’ll get through this…it will be alright and you’ll turn out fine”
	Be resilient and take risks	4	“Stay strong, don’t give up…be brave”
	Be kind to yourself	2	“Make sure you are your own best friend first”

## Discussion

The aim of the current study was to conduct an initial exploration of 2 of the priority research questions set by leading UK charity, Headlines: (1) What are the long-term physical and psychological effects for individuals with syndromic and non-syndromic craniosynostosis? and (2) Are individuals with craniosynostosis likely to suffer from mental health difficulties, or are they more resilient? Findings are discussed below in the context of current literature, and recommendations for future research and practice are made.

### Synthesis of Findings

On average, adults who participated in this study were less satisfied with their appearance, were more afraid of others judging them based on appearance, and felt more pressure to conform to societal appearance ‘ideals’ than adults without craniosynostosis. Participants reported these appearance concerns to have a negative impact on their self-esteem, romantic relationships, employment opportunities, and identity. Although 2 previous studies found adults with syndromic and non-syndromic craniosynostosis to be generally satisfied with their appearance (Raposo-Amaral et al., 2012; Salokorpi et al., 2019), findings from the current study align with research from the wider field of health psychology, which indicates that adults with appearance-altering conditions are liable to struggle with appearance pressures and other people's reactions, irrespective of the objective ‘severity’ of the condition (Rumsey and Harcourt, 2012). Strategies such as hiding affected features can be useful in the short-term, but the underlying psychological discomfort is likely to persist (Rumsey and Harcourt, 2012). Equally, seeking a more permanent change in appearance through surgery and/or other treatment can enhance well-being, but can also trigger individuals to question their sense of self or identity (Stavropoulos et al., 2011; Myhre et al., 2021). In line with broader research, the data therefore suggest that psychological interventions could seek to address appearance-related anxiety using evidence-based approaches, preferably starting in early adolescence (eg, Clarke et al., 2013).

Participants reported similar levels of social anxiety and social competence to the general population. However, qualitative responses highlighted difficulties with ‘fitting in’ and explaining craniosynostosis to others, which invoked anxiety in certain social situations and made initiating relationships problematic for some. Previous research has described how having a health condition, particularly one that is visible to others, can result in fewer friendships and romantic relationships (Stavropoulos et al., 2011; Tovetjärn et al., 2012; Fischer et al., 2014; Sakamoto et al., 2021). When discussing adult relationships, participants reported anxiety about being abandoned or unloved, as well as discomfort with closeness and intimacy, and a lack of belief that other people can be relied upon. Similar findings were identified in a recent survey of adults with cleft lip and/or palate using the same measures (Ardouin et al., 2021b), and together suggest this could be a particular challenge for adults with craniofacial conditions. However, participants also stated they actively used positive coping strategies to overcome social barriers, such as conversational skills, positive body language, and explaining their condition to others; a finding replicated in other studies of craniosynostosis (Stavropoulos et al., 2011; Raposo-Amaral et al., 2012). Given the importance of social connection in people's lives, psychological interventions could therefore focus on developing helpful coping strategies and social skills from a young age (Kapp-Simon et al., 2005).

For most participants, the recurrence risk of craniosynostosis was a cause for concern in relation to starting a family. This has also been identified in other studies, particularly in the case of syndromic diagnoses (O’Hanlon et al., 2012; Phipps and Skirton, 2017) and may offer some explanation for the observed higher rate of childlessness within the sample. Current understanding of the genetic and environmental causes of craniosynostosis also remains limited, and therefore so does the information available to young adults (Wilkie et al., 2017). However, some participants did not believe the recurrence risk to be a significant deterrent and felt they could cope if their child was born with craniosynostosis. One study of adults born with cleft lip and/or palate found adults’ attitudes toward recurrence to be largely determined by their own psychological adjustment [Stock and Rumsey, 2015], suggesting that if young people are supported to cope well with the challenges they are presented with as they grow, they will view craniosynostosis more positively in adulthood and feel more at ease about starting a family of their own.

Reported experiences of employment were mixed, with some describing few difficulties in work settings and others stating that their condition impacted their work life considerably. Although the UK Equality Act (2010) provides legal rights for people with physical disabilities and appearance-altering conditions, discrimination remains common in the workplace (Changing Faces, 2017) and within society more broadly (Roberts, 2014). Taken together, these findings suggest that improved awareness among both employers and employees may help to reduce stigma, while more targeted interventions to increase adults’ confidence when applying for and entering employment may be helpful for the individual.

Adults participating in the current study reported a range of physical health concerns and treatment needs, alongside a lack of awareness of craniosynostosis among non-specialist health professionals, such as General Practitioners. Combined with the lack of follow-up in adulthood, this could lead to difficulties accessing appropriate information and treatment, with participants reportedly being left to self-manage their physical and psychological health. Similar findings have been reported previously among adults with cleft lip and palate and other craniofacial conditions (Tovetjärn et al., 2012; Ardouin et al., 2021a), and suggest that a large number of adults may be receiving inappropriate treatment, no treatment at all, and/or facing long and frustrating referral times. While health professionals cannot be expected to possess specialist knowledge on every condition they treat, access to educational materials when needed may provide a broad understanding of craniosynostosis and reduce uncertainty surrounding the best approach to care (Stock and Costa, 2020), with the ultimate aim of improving adults’ treatment experiences.

Finally, adults with craniosynostosis reported more symptoms of general anxiety than norms, in addition to lower levels of optimism and resilience. Given that today's adults did not have access to specialist care or psychological support while growing up, they may be more prone to psychological distress. However, participants also offered advice to others with craniosynostosis, all of which was positively framed and indicative of self-care practices. In light of the view that optimism is a skill that can be learned (Seligman, 1991), the current study lends further support for examination of interventions aimed at increasing optimism and resilience within the context of craniofacial conditions. Future research that replicates the present study and others like it will be important to determine whether and how the current organization of UK care for craniosynostosis has led to improvements.

### Associations Between Outcomes and Psychological Domains

Almost all outcomes were correlated to some degree in the current study. In particular, global self-worth was strongly associated with a range of outcomes, including body esteem, fear of negative appearance evaluation, perceived sociocultural pressures, social anxiety, general anxiety, depressive symptoms, optimism, and resilience. This indicates that interventions targeted at increasing self-worth could have a potential protective effect across a range of psychological domains, making it a particularly important variable for further investigation.

Outcome variables were also categorized into 3 core domains of psychological adjustment: emotional (Global Self-Worth, HADS, LOT-R, CD-RISC10 and SPIN), social (Sociability, Intimacy and RAAS), and appearance-related (BES-AA, FNAES and PSPS). Findings suggest a strong association between emotional adjustment and appearance, between emotional adjustment and social adjustment, and to a lesser extent, between appearance and social adjustment. Previous craniofacial studies have also found associations between the different domains of psychological adjustment (eg, Roberts and Mathias, 2013; Feragen et al., 2015; Costa et al., 2021). Taken together, these findings suggest that intervening in one domain may indirectly improve adjustment in other domains. Further work is needed to confirm these associations and to assess the utility of this approach for psychological intervention.

### Future Directions

Supported by previous research in the field of cleft and craniofacial conditions, this small-scale study has illustrated the potential long-term physical and psychological impact of craniosynostosis. Treatment needs may continue into adulthood, particularly in the case of those who received care prior to the integration of psychologists within specialist units. Commissioning support for adults with ongoing healthcare needs should be considered, in addition to an improved awareness among non-specialists to improve the patient experience and the timeliness and relevance of referrals. Psychological support needs in adulthood may include coping with appearance-related anxiety, initiating and maintaining friendships and romantic relationships, family planning, support in the workplace, and resilience-building. Future research should aim to further develop the evidence base for psychological intervention. In the meantime, health professionals could consider a variety of ways in which they can support their patients’ psychological and holistic needs (Stock et al., 2020b).

### Methodological Considerations

This study represents an initial enquiry into 2 research priorities set by patient and parent representatives and health professionals in the area of craniosynostosis. The survey was largely based on robust, standardized measures consistent with other studies in the field. Along with the additional qualitative data collected, the survey allowed for key psychological and physical impacts of craniosynostosis in adulthood to be identified and preliminary recommendations for next steps to be made.

Nonetheless, a number of methodological limitations must be acknowledged. First, the sample obtained remained relatively small and, crucially, heterogenous. This made further analyses to investigate demographic and treatment-related variables unfeasible, and prevented the possibility of identifying predictors of psychological adjustment. Despite large effect sizes, the findings of this study should therefore be interpreted with caution and replicated using larger samples in future. Few differences between syndromic and non-syndromic cases were detected, which is contrary to previous literature (Feragen and Stock, 2017) and which could be attributed to a lack of power. While a greater impact of a syndrome should not be presumed, larger samples are needed to effectively examine potential variations across subgroups, including any additional impacts of cognitive impairment and other associated conditions (Feragen et al., 2014). Additionally, some participants were unaware of their exact diagnosis, and without medical records this makes the data more difficult to interpret. Future studies should also seek to include a matched control group where possible, rather than to rely upon general population norm data alone. Last, participants were predominantly recruited through Headlines and therefore cannot be assumed to be representative of the UK adult craniosynostosis population as a whole. Adults are a challenging group to recruit to research, since they are unlikely to be engaged in craniofacial treatment (Ardouin et al., 2021a). This is possibly most applicable to certain subgroups, such as those with less complex synostosis, who may have received little treatment since infancy and/or who may not identify with the diagnosis. Although online surveys attempt to bridge this gap and reach more individuals, there may have been several selection factors that narrow the generalizability of the results.

## Conclusions

This initial investigative study illustrates the potential long-term physical and psychological implications for individuals born with craniosynostosis. Improved treatment protocols are needed to address physical health concerns in adulthood, while dedicated psychological resources are necessary to promote emotional well-being, social confidence, and resilience throughout the lifespan. Future studies should include large-scale qualitative investigation, alongside combined efforts to achieve representative quantitative data allowing for investigation of predictor variables, demographic variables, and subtype analyses. Additional research aimed at improving understanding of craniosynostosis in primary care is warranted. Finally, further development of the evidence base for psychological intervention is needed, from the point of diagnosis onward.
